# Text feature extraction based on deep learning: a review

**DOI:** 10.1186/s13638-017-0993-1

**Published:** 2017-12-15

**Authors:** Hong Liang, Xiao Sun, Yunlei Sun, Yuan Gao

**Affiliations:** College of Computer and Communication Engineering, China University of Petroleum (East China), No. 66, Changjiang West Road, Huangdao District, Qingdao, 266580 China

**Keywords:** Deep learning, Feature extraction, Text characteristic, Natural language processing, Text mining

## Abstract

Selection of text feature item is a basic and important matter for text mining and information retrieval. Traditional methods of feature extraction require handcrafted features. To hand-design, an effective feature is a lengthy process, but aiming at new applications, deep learning enables to acquire new effective feature representation from training data. As a new feature extraction method, deep learning has made achievements in text mining. The major difference between deep learning and conventional methods is that deep learning automatically learns features from big data, instead of adopting handcrafted features, which mainly depends on priori knowledge of designers and is highly impossible to take the advantage of big data. Deep learning can automatically learn feature representation from big data, including millions of parameters. This thesis outlines the common methods used in text feature extraction first, and then expands frequently used deep learning methods in text feature extraction and its applications, and forecasts the application of deep learning in feature extraction.

## Review

### Introduction

Machine learning is a branch of artificial intelligence, and in many cases, almost becomes the pronoun of artificial intelligence. Machine learning systems are used to identify objects in images, transcribe speech into text, match news items, posts or products with users’ interests, and select relevant results of search [1]. Increasingly, these applications that are made to use of a class of techniques are called deep learning [1, 2]. Conventional machine learning techniques were limited in processing natural data in their raw form [1, 2].

For decades, constructing a pattern recognition or machine learning system required a careful engineering and considerable domain expertise to design a feature extractor that transformed the raw data (such as the pixel values of an image) into a suitable internal representation or feature vector which the learning subsystem, often a classifier, could detect or classify patterns in the input [1]. Representation learning is a set of methods that allow a machine to be given with raw data and to automatically discover the representations needed for detection or classification [1]. Deep learning methods are representation learning methods with multiple levels of representation, obtained by composing simply but nonlinear modules that each transforms the representation at one level (starting with the raw input) into a higher representation slightly more abstract level, with the composition of enough such transformations, and very complex functions can be learned [1, 2].

Text feature extraction that extracts text information is an extraction to represent a text message, it is the basis of a large number of text processing [3]. The basic unit of the feature is called text features [4]. Selecting a set of features from some effective ways to reduce the dimension of feature space, the purpose of this process is called feature extraction [5]. During feature extraction, uncorrelated or superfluous features will be deleted. As a method of data preprocessing of learning algorithm, feature extraction can better improve the accuracy of learning algorithm and shorten the time. Selection from the document part can reflect the information on the content words, and the calculation of weight is called the text feature extraction [5]. Common methods of text feature extraction include filtration, fusion, mapping, and clustering method. Traditional methods of feature extraction require handcrafted features. To hand-design an effective feature is a lengthy process, and deep learning can be aimed at new applications and quickly acquire new effective characteristic representation from training data.

The key aspect of deep learning is that these layers of features are not designed by human engineers, they are learned from data using a general purpose learning procedure [1]. Deep learning requires very little engineering by hand, so it can easily take advantage of the increase in the amount of available computation and data [1]. Deep learning has the advantage of identifying a model of unstructured data, and most people are familiar with the media such as images, sound, video, and text, all belonging to such data. Deep learning has produced extremely promising results for various tasks in natural language understanding [6] particularly topic classification, sentiment analysis, question answering [7], and language translation [2, 8, 9]. Its deep architecture nature grants deep learning the possibility of solving much more complicated AI tasks (Bengio, [42]) [2]. At present, deep learning feature representation includes autoencoder, restricted Boltzmann model, deep belief network, convolutional neural network and recurrent neural network, etc.

This thesis outlines the common methods used in text feature extraction first, and then expands frequently used deep learning methods in text feature extraction and its applications, and forecasts the application of deep learning in feature extraction. The main contribution of this work can be presented as follows:

- By reading a large amount of literature, the text feature extraction method and deep learning method is summarized
- A large amount of literature has been collected to summarize most of the application of the present text feature extraction method
- Summarized the most application of deep learning in text feature extraction
- The application of deep learning method in text feature extraction is prospected and summarized.

The rest of this paper is organized as follows: In Section 2, we introduce the text feature extraction method and its application in detail. Section 2 introduces the deep learning method and its application in text feature extraction and summarizes it in Section 3.

### Text feature extraction methods

Text feature extraction plays a crucial role in text classification, directly influencing the accuracy of text classification [3, 10]. It is based on VSM (vector space model, VSM), in which a text is viewed as a dot in N-dimensional space. Datum of each dimension of the dot represents one (digitized) feature of the text. And the text features usually use a keyword set. It means that on the basis of a group of predefined keywords, we compute weights of the words in the text by certain methods and then form a digital vector, which is the feature vector of the text [10]. Existing text feature extraction methods include filtration, fusion, mapping, and clustering method, which are briefly outlined below.

#### Filtering method

Filtration is quickly and particularly suitable for large-scale text feature extraction. Filtration of text feature extraction mainly has word frequency, information gain, and mutual information method, etc.

1. Word frequency
  Word frequency refers to the number of times that a word appears in a text. Feature selection through word frequency means to delete the words, whose frequencies are less than a certain threshold, to reduce the dimensionality of feature space. This method is based on such a hypothesis; words with small frequencies have little impact on filtration [3, 11, 12]. However, in the studies of information retrieval, it is believed that sometimes words with less frequency of occurrences have more information. Therefore, it is inappropriate to delete a great number of words simply based on the word frequency in the process of feature selection [11, 12].
2. Mutual information
  MI (mutual information) [13, 14] used for mutuality measurement of two objects is a common method in the analysis of computational linguistics models. It is employed to measure differentiation of features to topics in filtration. The definition of mutual information is similar to the one that of cross entropy. Mutual information, originally a concept in information theory, is applied to represent relationships between information and the statistical measurement of correlation of two random variables [13, 14]. Using mutual information theory for feature extraction is based on a hypothesis that words have big frequencies in a certain class but small in others, and the class has relatively large mutual information. Usually, mutual information is used as the measurement between a feature word and a class, and if the feature word belongs to the class, they have the largest amounts of mutual information. Since this method does not require any hypotheses on the property of relationship between feature words and classes, it is exceedingly suitable for the registration of features of text classification and classes [14].
  Time complexity of mutual information computation is similar to information gain. Its mean value is information gain. The deficiency of mutual information is that the score is extremely impacted by marginal probabilities of words [13, 14].
3. Information gain
  IG (information gain) is a common method for machine learning. In filtration, it is utilized to measure whether a known feature appears in a text of a certain relevant topic and how much predicted information of the topic. By computing information gain, features that frequently occur in positive samples instead of negative ones or the other way around can be obtained [15, 16].
  Information gain, an evaluation method based on entropy, involves lots of mathematical theories and complex theories and formulas about entropy. It is defined as the amount of information that a certain feature item is able to provide for the whole classification, taking no account of the entropy of any features but the difference value of entropy of the feature [17]. According to the training data, it computes information gain of each feature item and deletes items with small information gain while the rest are ranked in a descending order based on information gain.
4. Application
  Reference [18] has proposed that DF (document frequency) is the most simple method than others, but is inefficient on making use of the words with the lowest rising frequency well; Reference [19] has pointed that IG (information gain) can reduce the dimension of vector space model by setting the threshold, but the problem is that it is too hard to set the appropriate threshold; Reference [20] has thought that the method MI can make the words with the lowest rising frequency get more points than by other methods, because it is good at doing these words. In reference [21], a survey on intelligent techniques for feature selection and classification techniques used of intrusion detection has been presented and discussed. In addition, a new feature selection algorithm called intelligent rule based on attribute selection algorithm and a novel classification algorithm named intelligent rule-based enhanced multi-class support vector machine have been proposed. In reference [22], to address low efficiency and poor accuracy of keyword extraction of traditional TF-IDF (term frequency-inverse document frequency) algorithm, a text keyword extraction method based on word frequency statistics is put forward. Experimental results show that TF-IDF algorithm based on word frequency statistics not only overmatches traditional TF-IDF algorithm in precision ratio, recall ratio, and F1 index in keyword extraction, but also enables to reduce the run time of keyword extraction efficiently. In reference [23], a feature extraction algorithm based on average word frequency of feature words within and outside the class is presented. This algorithm can improve the classification efficiently. In reference [24], a modified text feature extraction algorithm is proposed. The experimental results suggest that this algorithm is able to describe text features more accurately and better be applied to text features processing, Web text data mining, and other fields of Chinese information processing. In reference [25], a method, which targets the feature of short texts and is able to automatically recognize feature words of short texts, is brought forward. According to experimental results, compared with traditional feature extraction methods, this method is more suitable for the classification of short texts. In reference [26], this paper presented an ensemble-based multi-filter feature selection method that combines the output of one third split of ranked important features of information gain, gain ratio, chi-squared, and ReliefF. The resulting output of the EMFFS is determined by combining the output of each filter method.

#### Fusion method

Fusion needs integration of specific classifiers, and the search needs to be conducted within an exponential increase interval. The time complexity is high [27, 28]. So, it is inappropriate to be used for feature extraction of large-scale texts [27, 28].

Weighting method is a special class of fusion. It gives each feature a weight within (0, 1) to train while making adjustments. Weighting method integrated by linear classifiers is highly efficient. K nearest neighbors (KNN) algorithm is a kind of learning method based on the instance [29].

1. Weighted KNN (K nearest neighbors)
  Han [30] put forward a kind of combination of KNN classifier weighted feature extraction problem. The method is for each classification of continuous cumulative values, and it has a good classification effect. KNN method as a kind of no parameters of a simple and effective method of text categorization based on the statistical pattern recognition performance outstanding; it can achieve higher classification accuracy rate and recall rate [29–31].
2. The center vector weighted method
  A weighted center vector classification method is proposed by Shankar [32], which firstly defines a method of characteristics to distinguish ability, the ability to distinguish between rights and get a new center vector. Algorithm requires multiple weighted methods (until the classification ability down).

#### Mapping method

Mapping has been widely applied to text classification and achieved good results [33]. It is commonly used to LSI (latent semantic index) [17] and PCA.

1. Latent semantic analysis
  LSA (latent semantic analysis) [17] (or LSI) was a new information retrieval algebraic model put forward by S.T. Dumais et al. in 1988. It is a computational theory or method that is used for knowledge acquisition and demonstration. It uses statistical computation method to analyze a mass of text sets, thereby extracts latent semantic structure between words, and employs this latent structure to represent words and texts so as to eliminate the correlation between words and reduce dimensionality by simplifying text vectors [17].
  The basic concept of latent semantic analysis is that mapping texts represented in high-dimensional VSM to lower dimensional latent semantic space. This mapping is achieved through SVD (singular value decomposition) of item or document matrix [19, 29].
  Application of LSA: information filtering, document index, video retrieval, text classification and clustering, image retrieval, information extraction, and so on.
2. Least squares mapping method
  Jeno [33] did a research on high-dimensional data reduction from the perspective of center vector and least squares. He believed dimensionality reduction has its predominance over SVD, because clustered center vectors reflect the structures of raw data, while SVD takes no account of these structures.
3. Application
  In reference [34], this study proposes a novel filter based on a probabilistic feature selection method, namely DFS (distinguishing feature selector), for text classification. The comparison is carried out for different datasets, classification algorithms, and success measures [34]. Experimental results explicitly indicate that DFS offers a competitive performance with respect to the abovementioned approaches in terms of classification accuracy, dimension reduction rate, and processing time [34].

#### Clustering method

Clustering takes the essential comparability of text features primarily to cluster text features into consideration. Then the center of each class is utilized to replace the features of that class. The advantage of this method is that it has a very low compression ratio, and basic accuracy of classification stays constant. Its disadvantage is the extremely high time complexity [35, 36].

1. CHI (chi-square) clustering method
  Through computation of each feature word’s contribution to each class (each feature word gets a CHI value to each class), CHI clustering clusters text feature words with the same contribution to classifications, making their common classification model replace the pattern that each word has the corresponding one-dimension in the conventional algorithm. The advantage of this method is relatively low time complexity [15, 16].
2. Concept Indexing
  In text classification, CI (concept indexing) [37] is a simple but efficient method of dimensionality reduction. By taking the center of each class as the base vector structure subspace (CI subspace), and then mapping each text vector to this subspace, the representation of text vectors to this subspace is acquired. The amount of classification included in training sets is exactly the dimensionality of CI subspace, which usually is smaller than that of the text vector space, so dimensionality reduction of vector space is achieved. Each class center as a generalization of text contexts in one classification can be considered as “concept,” and the mapping process of text vector can be regarded as a process of indexing in this concept space [38].
3. Applications
  In Reference [39], the method CHI is based on *χ* ^2^ distribution; if the distribution has been destroyed, the reliability of the low frequency may be declined. In Reference [40], the authors have described two approaches for combining the large feature spaces to efficient numbers using genetic algorithm and fuzzy clustering techniques. Finally, the classification of patterns has been achieved by using adaptive neuro-fuzzy techniques. The aim of the entire work is to implement the recognition scheme for classification of tumor lesions appeared in the human brain as space-occupying lesions identified by CT and MR images.

### Deep learning approach

Deep learning put forward by Hinton et al. in 2006 was a class of unsupervised learning [41]. Its concept comes from the studies of artificial neural network. Multi-layer perceptron with multiple implicit strata is a deep learning structure. By combining lower level features to form more abstract, higher level representing property classifications or features, deep learning is to discover distributed feature representation of data [2].

Deep learning as opposed to a surface learning, now a lot of learning methods are surface structure algorithm, and they exist some limitations, such as in the case of limited samples of complex function ability is limited, its generalization ability for complex classification problem is restricted by a certain [42]. Deep learning is by learning a kind of deep nonlinear network structure and implementing complex function approximation, according to the characterization of the input data distributed, and in the case of sample set, the essence characteristic of the data set [63] is seldom studied. The major difference between deep learning and traditional pattern recognition methods is that deep learning automatically learns features from big data, instead of adopting handcrafted features [2]. In the history of the development of computer vision, only one widely recognized good feature emerged in 5 to 10 years. But aiming at new applications, deep learning is able to quickly acquire new effective feature representation from training data.

Deep learning technology is applied in common NLP (natural language processing) tasks, such as semantic parsing [43], information retrieval [44, 45], semantic role labeling [46, 47], sentimental analysis [48], question answering [49–52], machine translation [53–56], text classification [57], summarization [58, 59], and text generation [60], as well as information extraction, including named entity recognition [61, 62], relation extraction [63–67], and event detection [68–70]. Convolution neural network and recurrent neural network are two popular models employed by this work [71].

Next, several deep learning methods, applications, improvement methods, and steps used for text feature extraction are introduced.

#### Autoencoder

An autoencoder, firstly introduced in Rumelhart et al. [72], is a feedforward network that can learn a compressed, distributed representation of data, usually with the goal of dimensionality reduction or manifold learning. An autoencoder usually has one hidden layer between input and output layer. Hidden layer usually has a more compact representation than input and output layers, i.e., hidden layer has fewer units than input or output layer. Input and output layer usually has the same setting, which allows an autoencoder to be trained unsupervised with same data fed in at the input and to be compared with what is at the output layer. The training process is the same as traditional neural network with backpropagation; the only difference lying in the error is computed by comparing the output to the data itself [2]. Mitchell et al. [73], showed a nice illustration of autoencoder. He built a three-layer structure (eight unit for input and output layer and three unit for the hidden layer in between), then he fed the one-hot vector representation into the input and output layer, the hidden layer turned out to approximating the data with inputs’ binary representation [2].

A stacked autoencoder is the deep counterpart of autoencoder and it can be built simply by stacking up layers. For every layer, its input is the learned representation of former layer and it learns a more compact representation of the existing learned representation. A stacked sparse autoencoder, discussed by Gravelines et al. [74], is stacked autoencoder where sparsity regularizations are introduced into the autoencoder to learn a sparse representation. A stacked denoising autoencoder, introduced by (Vincent et al. [75]) is an autoencoder where the data at input layer is replaced by noised data while the data at output layer stays the same; therefore, the autoencoder can be trained with much more generalization power [1].

In reference [76], for the characteristics of short texts, a feature extraction and clustering algorithm based on deep noise autoencoder is brought forward. This algorithm converts spatial vectors of high-dimensional, sparse short texts into new, lower-dimensional, substantive feature spaces by using deep learning network. According to experimental results, applying extractive text features to short text clustering significantly improves clustering effect and efficiently addresses high-dimensional and sparse short text space vectors. In reference [77], it is put forward by using sparse autoencoder of “deep learning” to automatically extract text features and combining deep belief networks to form SD (standard deviation) algorithm to classify texts. Experiments show that in the situation of fewer training sets, classification performance of SD algorithm is lower than that of traditional SVM (support vector machine), but when processing high-dimensional data, SD algorithm has a higher accuracy rate and recall rate than that compared with SVM. In reference [78], this paper presents the use of unsupervised pre-training using autoencoder with deep ConvNet in order to recognize handwritten Bangla digits. The proposed approach achieves 99.50% accuracy, which is so far the best for recognizing handwritten Bangla digits. In reference [79], human motion data is high-dimensional time-series data, and it usually contains measurement error and noise. In experiments, we compared the using of the row data and three types of feature extraction methods—principal component analysis, a shallow sparse autoencoder, and a deep sparse autoencoder—for pattern recognition [79]. The proposed method, application of a deep sparse autoencoder, thus enabled higher recognition accuracy, better generalization, and more stability than that which could be achieved with the other methods [79].

#### Restricted Boltzmann machine

**Fig. 1:**
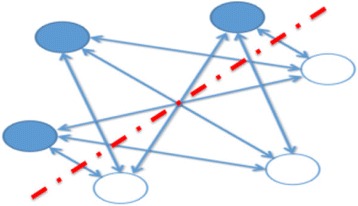
Illustration of RBM

RBM (restricted Boltzmann machine), originally known as Harmonium when invented by Smolensky [80], is a version of Boltzmann machine with a restriction that there are no connections either between visible units or between hidden units [2].This network is composed of visible units (correspondingly, visible vectors, i.e., data sample) and some hidden units (correspondingly hidden vectors). Visible vector and hidden vector are binary vectors, that is, their states take {0, 1}. The whole system is a bipartite graph. Edges only exist between visible units and hidden units, and there are no edge connections between visible units and between hidden units (Fig. 1).

Training process automatically requests for the repetition of the following three steps:

- During the forward transitive process, each input combines with a single weight and bias, and the result is transmitted to the hidden layer.
- During the backward process, each activation combines with a single weight and bias, and the result is transmitted to the visible layer for reconstruction.
- In the visible layer, KL divergence is utilized to compare reconstruction and initial input to decide the resulting quality.

Using different weights and biases repeating steps a–c until reconstruction and input are close as far as possible.

In reference [81], RBM is a new type of machine learning tool with strong power of representation, has been utilized as the feature extractor in a large variety of classification problems [81]. In this paper, we use the RBM to extract discriminative low-dimensional features from raw data with dimension up to 324 and then use the extracted features as the input of SVM for regression. Experimental results indicate that our approach for stock price prediction has great improvement in terms of low forecasting errors compared with SVM using raw data. In reference [82], this paper presents a deep belief networks (DBN) model and a multi-modality feature extraction method to extend features’ dimensionalities of short text for Chinese microblogging sentiment classification. The results demonstrate that, with proper structure and parameter, the performance of the proposed deep learning method on sentiment classification is better than the state-of-the-art surface learning models such as SVM or NB, which proves that DBN is suitable for short-length document classification with the proposed feature dimensionality extension method [82].

#### Deep belief network

DBN (deep belief networks) is introduced by Hinton et al. [83], when he showed that RBMs can be stacked and trained in a greedy manner [2]. DBN in terms of network structure can be regarded as a matter of stack, one of the restricted Boltzmann machine visible in the hidden layer is a layer on the layers.

**Fig. 2:**
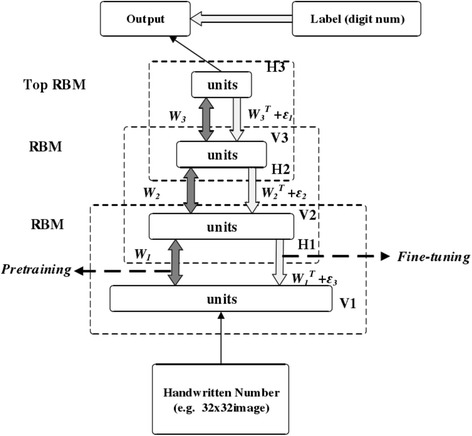
DBN network structures

Classical DBN network structure is a deep neural network constituted by RBM of some layers and BP of one layer. Figure 2 is the DBN network structure constituted by three RBM networks. Training process of DBN includes two phases: the first step is layer-wise pre-training, and the second step is fine-tuning [2, 84].

The process of DBN’s training model is primarily divided into two steps:

- Train RBM network of each layer respectively and solely under no supervision and ensure that as feature vectors are mapped to different feature spaces, and feature information is retained as much as possible.
- Set BP network at the last layer of DBN, receive RBM’s output feature vectors as its input feature vectors and train entity relationship classifier under supervision. RBM network of each layer is merely able to ensure that weights in its own layer to feature vectors of this layer instead of feature vectors of the whole DBN to be optimized. Therefore, a back propagation network propagates error information top-down to each layer of RBM and fine-tunes the whole DBN network. The process of RBM network training model can be considered as initialization of weight parameters of a deep BP network. It enables DBN to overcome a weakness that initialization of weight parameters of a deep BP network easily leads to local optimum and long training time.

Step 1 of the model above is called pre-training in deep learning’s terminology, and step 2 is called fine-tuning. Any classifiers based on specific application domain can be used in the layer with supervised learning. It does have to be BP networks [16, 84].

In reference [85], a novel text classification approach is proposed in this paper based on deep belief network. The proposed method outperforms traditional classifier based on the support of vector machine. Detailed experiments are also made to show the effect of different fine-tuning strategies and network structures on the performance of deep belief network [85]. Reference [86] proposed a biomedical domain-specific word embedding model by incorporating stem, chunk, and entity information and used them for DBN-based DDI extraction and RNN (recurrent neural network)-based gene mention extraction. In reference [87], this paper proposes a novel hybrid text classification model based on the deep belief network and softmax regression. The experimental results on Reuters-21578 and 20 Newsgroup corpus show that the proposed model can converge at the fine-tuning stage and perform significantly better than the classical algorithms, such as SVM and KNN [87].

#### Convolutional neural network

CNN (convolution neural network) [88] is developed in recent years and caused extensive attention of a highly efficient identification method. In the 1960s, Hubel and Wiesel, based on the research of the cat’s visual cortex cells, put forward the concept of receptive field [88]. Inspired, Fukushima made neurocognitive suggestions in the first implementation of CNN network and also felt that wild concept is firstly applied in the field of artificial neural network [89]. Then, in LeCun et al., the design and implementation is based on the error gradient algorithm training in the convolutional neural network [87, 88], and in some pattern recognition task set, the leading performance is relative to the other methods. Now, in the field of image recognition, CNN has become a highly efficient method of identification [90].

CNN is a multi-layer neural network; each layer is composed of multiple 2D surfaces, and each plane is composed of multiple independent neurons [91].A group of local unit is the next layer in the upper adjacent unit of input; this views local connection originating in perceptron [92, 93].

CNN is one of the artificial neural networks, with its strong adaptability and good at mining data local characteristics. The weights of sharing network structure make it more similar to the biological neural networks, reduce the complexity of the network model, a reduction in the number of weights, makes the CNN be applied in various fields of pattern recognition, and achieved very good results [94, 95]. CNN by combining local perception area, sharing the weight, the drop in space or time sampling to make full use of the data itself contains features such as locality, optimize network structure, and to ensure a degree of displacement invariability [93]. Through years of research work, the application of CNN is much more, such as face detection [96], document analysis [97], speech detection [98], and license plate recognition [99]. Kussul in 2006 was put forward by using permutation encoding technology of neural network in face recognition, handwritten digital recognition [100], and small object recognition tasks were made with some special performance of the classification system. And in 2012, the researchers implemented consecutive frames in the video data as a convolution of the neural network input data, so that one can introduce the data on the time dimension, so as to identify the motion of the human body [93, 101].

Relatively, typical automatic machine translation system automatically translate given words, phrases, and sentences into another language. Automatic machine translation made its appearance a long time ago, but deep learning has achieved great performance in two aspects: automatic translation of words and words in images. Word translation does not require any preprocessing of text sequence, and it can let algorithms learn the altered rules and altered afterwords are translated. Multi-layer large LSTM (long short-term memory, LSTM) RNNs are applied to this sort of translation. CNNs are used to determine images’ letters and their location. Once these two things were determined, the system would start to translate articles contained in the images into another language. It is usually called instant visual translation.

The description is of feature extraction in text categorization of several typical application of CNN model. In reference [102], sketched several typical CNN models are applied to feature extraction in text classification, and filter with different lengths, which are used to convolve text matrix. Widths of the filters equal to the lengths of word vectors. Then max pooling is employed to operate extractive vectors of every filter. Finally, each filter corresponds to a digit and connects these filters to obtain a vector representing this sentence, on which the final prediction is based. In reference [103], the model that is used is relatively complicated, in which convolution operation of each layer is followed by a max pooling operation. In reference [104], CNN convolves and abstracts word vectors of the original text with filters of a certain length, and thus previous pure word vector become convolved abstract sequences. At the end, LSTM is also used to encode original sentences. Its classification effect works better than that of LSTM. So here, CNN can be interpreted that it plays a role in feature extraction. In reference [105], LSTM unites with CNN. Vectorization representation of the whole sentence is gained, and prediction is made at the end. In reference [106], the model just slightly modifies the model above, but before convolution, it goes through a highway. In reference [107], the combined CNNs with dynamical systems to model physiological time series for the prediction of patient prognostic status were developed.

#### Recurrent neural network

**Fig. 3:**
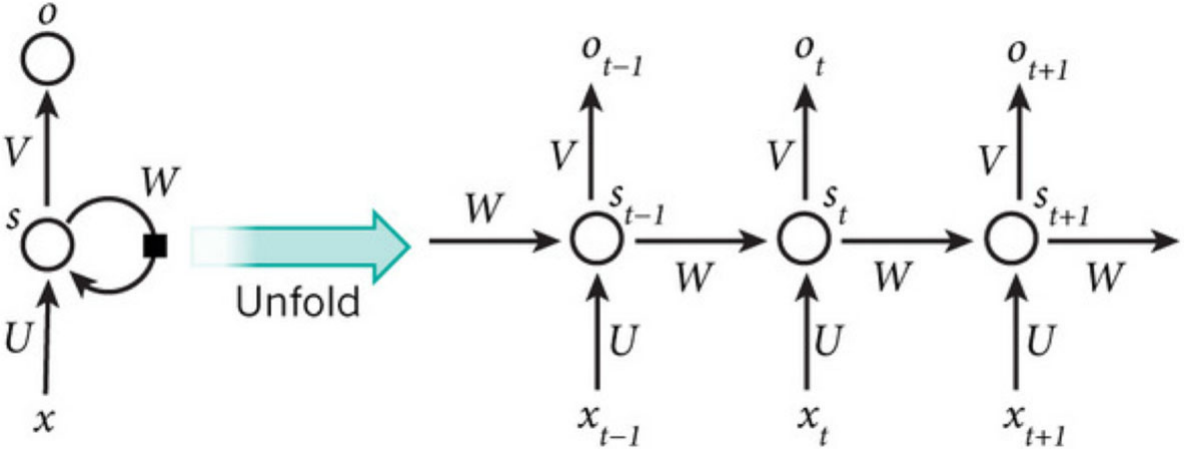
RNNs unflods in time

RNNs are used to process sequential data. In traditional neural network models, it is operated from the input layer to hidden layer to output layer. These layers are fully connected, and there is no connection between nodes of each layer. For tasks that involve sequential inputs, such as speech and language, it is often better to use RNNs (Fig. 3) [2]. RNNs processed an input sequence one element at a time, maintaining in their hidden units of a “state vector” that implicitly contains information about the history of all the past elements of the sequence. When we consider the outputs of the hidden units at different discrete time steps as if they were the outputs of different neurons in a deep multi-layer network, it becomes clear how we can apply backpropagation to train RNN [2].

RNNs are very powerful dynamic systems, but training them has proved to be problematic because the backpropagated gradients either grow or shrink at each step, many times the steps typically explode or vanish [108, 109].

The artificial neurons (for example, hidden units grouped under nodes with values *s*_t_ at time *t*) get inputs from other neurons at previous time steps (this is represented with the black square, representing a delay of one time step, on the left). In this way, a recurrent neural network can map an input sequence with elements *x*_t_ into an output sequence with elements *o*_t_, with each *o*_t_ depending on all the previous *x*_t′_ (for *t*′ ≤ t) [2]. The same parameters (matrices U, V, W) are used at each time step. Other architecture is possible, including a variant in which the network can generate a sequence of outputs (for example, words), each of which is used as inputs for the next time step. The backpropagation algorithm (Fig. 1) can be directly applied to the computational graph of the unfolded network on the right, to compute the derivative of a total error (for example, the log probability of generating the right sequence of outputs) with respect to all the states *s*_t_ and all the parameters [2].

There are several improved RNN, such as simple RNN (SRNs), bidirectional RNN, deep (bidirectional) RNN, echo state networks, Gated Recurrent Unit RNNs, and clockwork RNN (CW-RNN.

Reference [110] extends the previously studied CRF-LSTM (conditional random field, long short-term memory) model with explicit modeling of pairwise potentials and also proposes an approximate version of skip-chain CRF inference with RNN potentials. This paper uses this method for structured prediction in order to improve the exact phrase detection of clinical entities. In Reference [111], a two-stage neural network architecture constructed by combining RNN with kernel feature extraction is proposed for stock prices forecasting. By examining the stock prices data, it is shown that RNN with feature extraction outperforms single RNN, and RNN with kernel performs better than those without kernel.

#### Others

Many references are related to the infrastructure techniques of deep learning and performance modeling methods.

In Reference [112], this study develops a total cost of ownership (TCO) model for flash storage devices and then plugs a Write Amplification (WA) model of NVMe SSDs we build based on the empirical data into this TCO model. Experimental results show that min-TCO can reduce the TCO and keep relatively high throughput and space utilization of the entire datacenter storage. In Reference [113], this study characterizes the performance of persistent storage option (through data volume) for I/O intensive, dockerized applications. This paper then proposes novel design guidelines for an optimal and fair operation of both homogeneous and heterogeneous environments mixed with different applications and workloads. In Reference [114], this study proposes a complete solution called “AutoReplica”—a replica manager in distributed caching and data processing systems with SSD-HDD tier storages. In Reference [115], this research proposes a performance approximation approach FiM to model the computing performance of iterative, multi-stage applications running on a master-compute framework. In Reference [116], this research designs a Global SSD Resource Management solution (GReM), which aims to fully utilize SSD resources as a second-level cache under the consideration of performance isolation. Experimental results show that GReM can capture the cross-VM IO changes to make correct decisions on resource allocation, and thus obtain high IO hit ratio and low IO management costs, compared with both traditional and state-of-the-art caching algorithms.

In terms of methodology, the paper uses the optimization methods in resource management which are also involved in some references.

In Reference [117], for this study, the techniques of virtual machine migration are understood, and the affected reduplications on migration are evaluated. From this study, grouping virtual machines based on similar elements improves the overhead from reduplications and compression but estimates which virtual machines are best grouped together. In Reference [118], this study designs new VMware Flash Resource Managers (vFRM and glb-vFRM) under the consideration of both performance and the incurred cost for managing flash resources. In Reference [119], this study aims to develop an efficient speculation framework for a heterogeneous cluster. The results show that this paper’s solution is efficient and effective when handling the speculative execution. The job execution time in our system is superior to that in the current Hadoop distribution. In Reference [120], this study investigates a potential attack from a compromised internal node against the overall system performance, also present a mitigation scheme that protects a Hadoop system from such attack. In Reference [121], the authors investigate a superior solution which ensures all branches acquire suitable resources according to their workload demand in order to let the finish time of each branch be as close as possible. The experiments demonstrate that the new scheduler effectively reduces the span and improves resource utilizations for these applications, compared to the current FIFO and FAIR schedulers. In Reference [122], this study investigates storage layer design in a heterogeneous system considering a new type of bundled jobs where the input data and associated application jobs are submitted in a bundle. The results show significant performance improvements in terms of execution time and data locality.

## Conclusion

Selection of text feature item is a basic and important matter for text mining and information retrieval. Feature extraction means that according to the certain feature extraction metrics, the extract is relevant to the original feature subsets from initial feature sets of test sets, so as to reduce the dimensionality of feature vector spaces. During feature extraction, the uncorrelated or superfluous features will be deleted. As a method of data preprocessing of the learning algorithm, feature extraction can better improve the accuracy of learning algorithm and shorten the time. Compared with other machine learning methods, deep learning is able to detect complicated interactions from features, learn lower level features from nearly unprocessed original data, mine charateristics that is not easy to be detected, hand class members with high cardinal numbers, and process untapped data.

Compared with the several other models of deep learning, the recurrent neural network has been widely applied in NLP but RNN is seldom used in text feature extraction, and the basic reason is that RNN mainly targets data with time sequence. Besides, generative adversarial network model, which was proposed by Ian J. Goodfellow [123] the first time in 2014, has achieved significant results in the field of deep learning generative model in a short period of 2 years. This thesis brings forward a new frame that can be used to estimate and generate a model in the opponent process and that be viewed as a breakthrough in unsupervised representation learning compared with previous algorithms. Now, it is mainly applied to generate natural images. But it has not made significant progress in text feature extraction.

There are some bottlenecks in deep learning. Both supervised perception and reinforcement learning need to be supported by large amounts of data. At present, we have the largest dataset of diabetes from 301 hospitals, which will support us to deal with medical problems with deep learning approach, so that we can better use deep learning approach in text feature extraction. Furthermore, they have a very bad performance on the advanced plan and only can do some simplest and the most direct pattern discrimination works. Volatile data quality results in unreliability, inaccuracy, and unfairness need improvement in the future. Owing to intrinsic characteristics of text feature extraction, every method has its own advantages as well as unsurmountable disadvantages. If possible, multiple extraction methods can be applied to extract the same feature.
